# Antiphospholipid antibodies and neurological manifestations in acute COVID-19: A single-centre cross-sectional study

**DOI:** 10.1016/j.eclinm.2021.101070

**Published:** 2021-08-12

**Authors:** Laura A. Benjamin, Ross W. Paterson, Rachel Moll, Charis Pericleous, Rachel Brown, Puja R. Mehta, Dilan Athauda, Oliver J. Ziff, Judith Heaney, Anna M. Checkley, Catherine F. Houlihan, Michael Chou, Amanda J. Heslegrave, Arvind Chandratheva, Benedict D. Michael, Kaj Blennow, Vinojini Vivekanandam, Alexander Foulkes, Catherine J. Mummery, Michael P. Lunn, Stephen Keddie, Moira J. Spyer, Tom Mckinnon, Melanie Hart, Francesco Carletti, Hans Rolf Jäger, Hadi Manji, Michael S. Zandi, David J. Werring, Eleni Nastouli, Robert Simister, Tom Solomon, Henrik Zetterberg, Jonathan M. Schott, Hannah Cohen, Maria Efthymiou, The UCLH Queen Square COVID-19 Biomarker Study group

**Affiliations:** a National Hospital for Neurology and Neurosurgery, University College London Hospitals NHS Foundation Trust, Queen Square, London WC1N 3BG, UK; b Laboratory of Molecular and Cell Biology, UCL, Gower St, Kings Cross, London WC1E 6BT, UK; c UCL Queen Square Institute of Neurology, London, UK; d Brain Infections Group, University of Liverpool, Liverpool, Merseyside, UK; e Darent Valley Hospital, Dartford, Kent, UK; f UK Dementia Research Institute, London, UK; g Haemostasis Research Unit, Department of Haematology, UCL, UK; h Department of Haematology, University College London Hospitals, UK; i Imperial College London, National Heart and Lung Institute, UK; j Department of Infection and Immunity, University College London, UK; k Francis Crick Institute, London, UK; l Department of Clinical Virology, University College London Hospitals NHS Foundation Trust, UK; m Advanced Pathogens Diagnostic Unit, University College London Hospitals NHS Foundation Trust, UK; n Hospital of Tropical Medicine, University College London Hospitals, UK; o Neuroimmunology and CSF Laboratory, National Hospital for Neurology and Neurosurgery, UK; p Veterinary and Ecological Sciences, National Institute for Health Research Health Protection Research Unit in Emerging and Zoonotic Infections, Institute of Infection, University of Liverpool, UK; q Walton Centre NHS Foundation Trust, Liverpool, UK; r Department of Psychiatry and Neurochemistry, Institute of Neuroscience and Physiology, the Sahlgrenska Academy at the University of Gothenburg, Mölndal, Sweden; s Department of Immunology and Inflammation, Imperial College London, UK; t Institute of Child Health, UCL, UK; u Stroke Research Centre, UCL Queen Square Institute of Neurology, London, UK

## Abstract

**Background:**

A high prevalence of antiphospholipid antibodies has been reported in case series of patients with neurological manifestations and COVID-19; however, the pathogenicity of antiphospholipid antibodies in COVID-19 neurology remains unclear.

**Methods:**

This single-centre cross-sectional study included 106 adult patients: 30 hospitalised COVID-neurological cases, 47 non-neurological COVID-hospitalised controls, and 29 COVID-non-hospitalised controls, recruited between March and July 2020. We evaluated nine antiphospholipid antibodies: anticardiolipin antibodies [aCL] IgA, IgM, IgG; anti-beta-2 glycoprotein-1 [aβ_2_GPI] IgA, IgM, IgG; anti-phosphatidylserine/prothrombin [aPS/PT] IgM, IgG; and anti-domain I β_2_GPI (aD1β2GPI) IgG.

**Findings:**

There was a high prevalence of antiphospholipid antibodies in the COVID-neurological (73.3%) and non-neurological COVID-hospitalised controls (76.6%) in contrast to the COVID-non-hospitalised controls (48.2%). aPS/PT IgG titres were significantly higher in the COVID-neurological group compared to both control groups (*p* < 0.001). Moderate-high titre of aPS/PT IgG was found in 2 out of 3 (67%) patients with acute disseminated encephalomyelitis [ADEM]. aPS/PT IgG titres negatively correlated with oxygen requirement (FiO_2_*R*=-0.15 *p* = 0.040) and was associated with venous thromboembolism (*p* = 0.043). In contrast, aCL IgA (*p* < 0.001) and IgG (*p* < 0.001) was associated with non-neurological COVID-hospitalised controls compared to the other groups and correlated positively with d-dimer and creatinine but negatively with FiO_2_.

**Interpretation:**

Our findings show that aPS/PT IgG is associated with COVID-19-associated ADEM. In contrast, aCL IgA and IgG are seen much more frequently in non-neurological hospitalised patients with COVID-19. Characterisation of antiphospholipid antibody persistence and potential longitudinal clinical impact are required to guide appropriate management.

**Funding:**

This work is supported by UCL Queen Square Biomedical Research Centre (BRC) and Moorfields BRC grants (#560441 and #557595). LB is supported by a Wellcome Trust Fellowship (222102/Z/20/Z). RWP is supported by an Alzheimer's Association Clinician Scientist Fellowship (AACSF-20-685780) and the UK Dementia Research Institute. KB is supported by the Swedish Research Council (#2017-00915) and the Swedish state under the agreement between the Swedish government and the County Councils, the ALF-agreement (#ALFGBG-715986). HZ is a Wallenberg Scholar supported by grants from the Swedish Research Council (#2018-02532), the European Research Council (#681712), Swedish State Support for Clinical Research (#ALFGBG-720931), the Alzheimer Drug Discovery Foundation (ADDF), USA (#201809-2016862), and theUK Dementia Research Institute at UCL. BDM is supported by grants from the MRC/UKRI (MR/V007181/1), MRC (MR/T028750/1) and Wellcome (ISSF201902/3). MSZ, MH and RS are supported by the UCL/UCLH NIHR Biomedical Research Centre and MSZ is supported by Queen Square National Brain Appeal.

**Research in context**

**Evidence before this study**

We searched PubMed for articles published up to April 23rd, 2021, using the keywords “coronavirus”, “COVID-19” and “antiphospholipid antibodies” “anticardiolipin”, “beta-2 glycoprotein-I “phosphatidylserine/prothrombin”, and “neurological manifestations”, with no language restriction. The review indicated that neurological manifestations were an important complication of COVID-19. A limited number of case series showed elevated antiphospholipid antibodies amongst patients with neurological complications in COVID-19, but no study has determined any pathogenic associations.

**Added value of this study**

Using nine antiphospholipid antibodies, we showed that anti-phosphatidylserine/prothrombin (aPS/PT) IgG was associated with COVID-associated neurological manifestations, specifically acute disseminated encephalomyelitis (ADEM). This antiphospholipid antibody was related to venous thromboembolism, and severity of respiratory disease in the absence of systemic inflammation, suggestive of respiratory microvascular thrombosis. In contrast, anticardiolipin antibodies IgA and IgG were associated with non-neurological COVID-hospitalised controls, and correlated with hypercoagulability, respiratory and renal disease. Antiphospholipid antibodies are ubiquitous in COVID-19 and might have specific pathological roles in COVID-19 and neurological manifestations of COVID-19.

**Implications of all the available evidence**

Our study provides the first biochemical evidence that implicates antiphospholipid antibodies in the neurological manifestations of COVID-19. Our preliminary findings show that aPS/PT IgG might be an important feature in COVID-19-associated ADEM, mediated by non-thrombotic mechanisms in the brain, but thrombotic mechanisms systemically. Characterisation of whether these antiphospholipid antibodies are persistent and their potential longitudinal clinical impact are required, in order to guide appropriate management.

Alt-text: Unlabelled box

## 1 Introduction

The severe acute respiratory syndrome coronavirus 2 (SARS‐CoV-2), causing COVID-19 is not just a viral pulmonary infection with life-threatening respiratory complications but a multiple-organ disorder accompanied by hypercoagulability [1]. Thromboembolic events involving arterial, venous and micro-circulation are commonly reported [2,3]. While sepsis-induced hypercoagulability is a recognised complication of severe respiratory infections, most patients with COVID-19 maintain normal concentrations of coagulation factors, with normal prothrombin time, and other coagulation screening tests, and platelet counts, suggesting that COVID-19 induces a unique prothrombotic state [4].

Antiphospholipid syndrome (APS) is an acquired autoimmune disorder with the potential for life-threatening complications [5]. Antiphospholipid antibodies (lupus anticoagulant [LA], IgG and/or IgM anti-beta-2 glycoprotein-1 [aβ_2_GP1] and anticardiolipin antibodies [aCL]) that persist for more than 12-weeks, are well-recognised causes of venous, arterial, microvascular thrombosis and/or pregnancy morbidity; cardinal features of APS [5]. These antiphospholipid antibodies, including those in criteria sets (LA, aCL and aβ_2_GPI) as well as non-criteria sets (antibodies to Domain 1 of β_2_GPI ([aβ_2_GPI-D1] and phosphatidylserine/prothrombin antibodies [aPS/PT]), can activate the endothelium, platelets and neutrophils, thereby shifting the blood-endothelium interface to a prothrombotic state [6]. Initial reports have demonstrated an increased frequency of antiphospholipid antibodies in COVID-19 patients and evidence of thrombosis *in vivo* [7]. However, the interpretation of these results remains unsubstantiated as transient antiphospholipid antibodies can also occur during viral infections [8].

The central nervous system is a major target of antiphospholipid antibodies [9]. Although thrombotic damage explains many of the neurological manifestations of APS, direct immune-mediated processes may also be involved [9]. The reason why patients develop different antiphospholipid antibody associated neurological symptoms is currently unknown. Possible explanations may relate to autoantibody classes or epitope targets. Neurological manifestations were reported in 36.4% of hospitalised COVID-19 patients in one study [10]. A limited number of case series have shown prevalent antiphospholipid antibodies in acute COVID-19 associated stroke [[11], [12], [13], [14], [15]]. However, these findings are only observational, and therefore, do not exclude the possibility that these prevalent antiphospholipid antibodies are incidental.

Catastrophic APS (CAPS) is a rare and severe form of APS, and associated with a high mortality rate [15]. The diagnostic criteria for CAPS include multiple organ failure developing over a short period, histopathological evidence of multiple small vessel occlusions and high titres of antiphospholipid antibodies [15]. In a CAPS registry, 56% of the reported cases developed neurological manifestations [16], suggesting that this may be a common complication of APS.

The extent to which antiphospholipid antibodies contribute to thrombotic or immune-mediated neurological manifestations in COVID-19 is unclear. This study aimed to estimate the burden and determine the associations and clinical correlations of antiphospholipid antibodies in COVID-19 populations, with and without neurological manifestations.

## 2 Methods

### 2.1 Study design and participants

We carried out a cross-sectional study of adult patients over 18 years of age who presented to University College London Hospital between March and June 2020 and met the European Centre for Disease Prevention and Control (ECDC) definition of COVID-19 [17]. Participants with new neurological signs or symptoms according to standardised definitions within 40 days of respiratory or systemic COVID-19 symptoms were defined as having a COVID-neurological illness [18]. Participants meeting the ECDC definition of COVID-19 but without neurological signs/symptoms or history of significant neurological disease were defined as COVID-hospitalised controls; they were hospitalised during the same period as the neurological cases. Participants with positive SARS-CoV-2 PCR who did not require hospital admission and had serology samples collected contemporaneously in a previously described study, represented COVID-non-hospitalised controls [19].

We further classified COVID-neurological cases as (1) 'central': encephalitis; encephalopathy; acute disseminated encephalomyelitis (ADEM) or stroke and other (including intracranial hypertension and a central pain syndrome); (2) 'peripheral': Guillain-Barré syndrome (GBS) or (3) other according to clinical consensus criteria as previously described [12].

Clinical data were extracted from electronic records (EPIC, Madison, WI, USA). Patient characteristics, including age, sex, ethnicity, major co-morbidities, laboratory studies, and treatments were recorded, as described previously [12]. Clinical outcomes, including venous thromboembolism outside the central nervous system, ITU admission and 28-day mortality, were determined based on the review of electronic notes in January 2021. Additional blood was collected alongside samples collected as part of standard clinical care with written informed consent. Consent was obtained according to the Declaration of Helsinki. The Queen Square ethics committee approved this study (12-LO-1540).

### 2.2 Procedure

We evaluated nine different antiphospholipid antibodies following the national and international guidelines[5,20,21] using Quanta Lite aCL IgG, aCL IgM, aCL IgA, aβ_2_GPI IgG, aβ_2_GPI IgM, aβ_2_GPI IgA, aPS/PT IgG, and aPS/PT IgM kits (Inova Diagnostics Inc.), according to the manufacturer's instructions [22]. Positivity for aCL was defined as greater than the 99th centile (>20 GPLU or MPLU), for aβ_2_GPI as greater than the 99th centile (>20 SGu or SMu) and for aPS/PT as greater than the 99th centile (˃30 IgG or IgM units).

Antibodies against domain I B_2_GPI (aD1β_2_GPI) IgG were established as previously reported [23]. Purified aD1β_2_GPI isolated from a patient's serum with APS was used as the calibrator, serially diluted to obtain a standard curve and arbitrary activity units assigned to each point. aD1β_2_GPI activity was defined as DI units (GDIU) and calculated as per aCL and aβ_2_GPI assays. aD1β_2_GPI positivity was defined as >99th percentile of the mean activity of 200 healthy individuals [23]. The cut-off that defined a moderate or high titre for aD1β_2_GPI was 10GDIU [moderate titre] and 20GDIU [high titre].

We also arbitrarily defined moderate titre as ≥40 units/ml, high titre as ≥80 units/ml for all antiphospholipid antibodies. Testing for lupus anticoagulant was not performed due to a lack of citrated plasma samples. The criteria set of antiphospholipid antibodies that contribute to the definition of APS included aB_2_GPI [IgM and IgG] and aCL [IgM and IgG].

### 2.3 Statistical analysis

Continuous variables were summarised using means and medians and compared using Student's independent-samples *t*-test or Mann-Whitney U test as appropriate. Assumption of normality was verified for the appropriate reporting of means. Furthermore, distributions were verified to be similar, for the assumptions of the Mann-Whitney U test to hold. Categorical data were presented as percentages and compared using a chi-squared test. Pearson's product-moment correlation analysed linear correlations between antiphospholipid antibodies and other markers. The strength and direction of relationships were measured using simple linear regression, examining residuals to ensure fulfilment of linear regression assumptions. All statistical analyses and graphs were generated using Stata 14 (College Station, TX, USA) and Prism 8.3.1 (GraphPad, La Jolla, California, USA); *p* < 0.05 was considered significant. We report medians and interquartile range(IQR).

### 2.4 Role of the funding source

The funder of the study had no role in study design, data collection, data analysis, data interpretation, or writing of the report. LAB, RP, and ME had full access to all data and the final responsibility to submit for publication.

## 3 Results

### 3.1 Participants

One hundred and six adult participants (30 COVID-neurological cases, 47 non-neurological COVID-hospitalised controls, 29 COVID-non-hospitalised controls) were included in this cross-sectional study. The median (IQR) age was 55-years (43, 62), of whom 39 (37%) were female. Demographic and clinical characteristics are presented in Table 1. The COVID-non-hospitalised controls had only demographic and no detailed illness data. They were presumed to be healthy with mild COVID-19 illness, and therefore, did not necessitate hospital admission. Amongst all clinical characteristics of hospitalised individuals, only World Health Organisation (WHO) COVID-19 severity classification, low molecular weight heparin (LMWH) treatment status, ITU admission, and 28-day mortality differed significantly between the COVID-hospitalised controls and COVID-neurological cases. The COVID-hospitalised controls compared with the COVID-neurological group had a more (WHO) severe illness (87% versus 32%; *p* < 0.001), were more likely to be on treatment dose LMWH (35% versus 4%; *p* = 0.003), admitted to ITU (83% versus 24%; *p* =< 0.001) and die by 28-days (26% versus 7%; *p* = 0.042). When comparing the COVID-non-hospitalised with the hospitalised group, the non-hospitalised controls were younger (median 43-years [38,52]<0.001), less frequently male (<0.05) and more likely to be white in ethnicity (<0.05) (Table 1).

**Table 1:** Demographic and clinical characteristics of patients with and without COVID neurological disease.

	COVID-Neurological (n = 30)	COVID Hospitalised (n = 47)	*p*	COVID non-hospitalised (n = 29)	*p*
Median Age (IQR), years	58 (47,65)	57 (48,65)	NS	43 (38,52)	<0.001
Male Sex	17 (59)	36 (77)	NS	13 (45)	<0.05
Non-white ethnicity	13 (46)	32 (68)	NS	12 (41)	<0.05
ECDC COVID-19 classification:				–	
Laboratory	21 (70)	36 (77)	NS		
Probable	8 (26)	8 (17)			
Possible	1 (3)	3 (6)			
WHO COVID-19 severity classification:				NA	
Mild/Moderate	19 (68)	6 (13)	<0.001		
Severe	9 (32)	41 (87)			
Co-morbidities					
Hypertension	5 (17)	15 (32)	NS	NA	
Diabetes	5 (17)	11 (23)	NS	NA	
Hypercholesterolaemia	6 (21)	6 (13)	NS	NA	
Malignancy	5 (18)	6 (13)	NS	NA	
Ischaemic heart disease	1 (3)	4 (9)	NS	NA	
Median Body Mass Index	25 (23,30)	25 (23,30)	NS	NA	
Antithrombotic treatment*					
LMWH				NA	
- No treatment	14 (52)	10 (22)	<0.01		
- Prophylactic	12 (44)	20 (43)			
- Therapeutic	1 (4)	16 (35)			
NOAC	2 (7)	4 (9)	NS	NA	
Antiplatelet	6 (21)	7 (17)	NS	NA	
Outcome					
Venous Thromboembolism	3 (13)	9 (19)	NS	NA	
28-day mortality	2 (7)	12 (26)	<0.05	NA	
ITU admission	7 (24)	38 (83)	<0.001	NA	
Median length of hospital admission	19 (5,65)	32 (21, 57)	NS		
Neurological Diagnoses					
Encephalopathy	9 (30)	NA		NA	
Encephalitis	3 (10)				
ADEM	3 (10)				
Stroke	5 (17)				
GBS	8 (27)				
Other	2 (6)				

⁎ one hospitalised COVID patient was on warfarin (INR 1.7), NOAC – Non-vitamin K oral anticoagulants (Apixaban). No patients were on prior anticoagulation; LMWH – Low molecular weight heparin. Figures in parentheses (represent percentage (%). NA: no available NS: not significant.

### 3.2 High prevalence of antiphospholipid antibodies in hospitalised COVID-19 patients

Seventy-two of the 106 patients (67.9%) tested positive for at least one type of antiphospholipid antibodies (Table 2).

**Table 2:** Prevalence of antiphospholipid antibodies across the COVID-neurological, hospitalised and non-hospitalised groups.

n (%) (median, range)	COVID Neurological *n* = 30	Hospitalised COVID *n* = 47	Non-hospitalised COVID *n* = 29
aβ_2_GPI IgG	4 (13.3%)	2 (4.3%)	1 (3.5%)
	(3.5, 1.9–34.2)	(3.2, 2.2–91.2)	(1.8, 1.1–8.3)
aβ_2_GPI IgM	2 (6.7%)	8 (17.0%)	0 (0%)
	(4.2, 1.8–26.1)	(5.0, 1.3–69.0)	(3.2,1.0–129.2)
aβ_2_GPI IgA	8 (26.7%)	12 (25.5%)	1 (3.5%)
	(7.1, 1.7–87.3)	(8.6, 3.2–150)	(4.1, 1.0–32.6)
aCL IgG	1 (3.3%)	10 (21.3%)	3 (10.3%)
	(8.7, 2.7–21.1)	(12.3, 4.5–79.0)	(8.5, 2.8–39.0)
aCL IgM	17 (56.7%)	24 (51.1%)	7 (24.1%)
	(21.3, 8.7–100.4)	(23.8, 8.9–184.6)	(16.0, 7.7–52.7)
aCL IgA	1 (3.3%)	10 (21.3%)	1 (3.5%)
	(5.4, 0.6–49.8)	(10.8, 2.4–79.0)	(6.6, 0.6–49.8)
aPS/PT IgG	3 (10%)	0 (0%)	0 (0%)
	(11.8, 5.6–123.4)	(8.4, 3.8–26.7)	(6.9, 4.7–27.4)
aPS/PT IgM	3 (10%)	5 (10.6%)	6 (20.7%)
	(12.7, 2.5–84.1)	(9.6, 2.2–80.1)	(21.0, 6.5–67.0)
aD1β2GPI IgG	3 (10%)	7(14.9%)	0 (0%)
	(7.5, 5.2–1000)	(6.6, 4.7–54.3)	(5.2, 4.7–9.5)
Any positive aPL	22 (73.3%)	36 (76.6%)	14 (48.2%)
Positive for at least 2 aPL	10 (33.3%)	22 (46.8%)	4 (13.8%)
Positive for at least ≥3 aPL	7 (23.3%)	13 (27.7%)	0 (0%)
Positive for one criteria aPL	16 (53.3%)	16 (34.0%)	9 (31.0%)
Positive for two criteria aPL	9 (30%)	9 (19.1%)	0 (0%)

aPL antiphospholipid antibodies; aCL, anticardiolipin antibodies; aB2GPI, anti–B_2_ glycoprotein I antibodies; aPS/PT, anti-phosphatidylserine/prothrombin antibodies; aD1β2GPI IgG, anti- Domain I B_2_ glycoprotein I antibody. Cut off values for antiphospholipid antibodies are based on the manufactures cut-off, and for Domain I 10 units/ml. *Criteria antiphospholipid antibody includes aCL IgM and IgG and aB_2_GPI IgM and IgG. Missing data Covid-Neuro *n* = 3, Covid-Hospitalised *n* = 0, Covid non-hospitalised *n* = 0.

In the COVID-neurological cohort, 22 (73.3%) were positive for at least one antiphospholipid antibody; 10 (33%) for at least two antiphospholipid antibodies and 7 (23.3%) for three or more. ACL IgM exhibited the highest prevalence (56.7%) followed by aB_2_GPI IgA (26.7%), aPS/PT IgG and IgM and then aD1β_2_GPI IgG (10%). One neurological patient had an unexpectedly high level of aD1β_2_GPI IgG (>1000 units/ml) and this case is described in Supplement Table 1. Notably, aPS/PT IgG antiphospholipid antibody had moderate-high titre levels only in the COVID-neurological group (Table 3). Overall, a total of 16 (53.3%) were positive for at least one antiphospholipid antibody used in the APS criteria (aB_2_GPI and aCL IgG and IgM) and 9 (30%) for two (Table 2). Furthermore, 11 (36.6%) COVID-neurological patients exhibited moderate (≥40 units) and 6 (20%) had high (≥80 units) titres.

**Table 3:** Proportion of neurological patients with moderate-high antiphospholipid antibody titre in COVID-19.

	Encephalopathy *N* = 9 (%)	Encephalitis *n* = 3 (%)	ADEM *n* = 3 (%)	Stroke *n* = 5 (%)	GBS *n* = 8 (%)	Other *n* = 2 (%)
aβ_2_GPI IgG	2 (22)	1 (33)	0	0	0	1 (50)
aβ_2_GPI IgA	4 (44)	1 (33)	0	1 (20)	2 (25)	0
aCL IgM	6 (67)	1 (33)	1 (33)	3 (80)	4 (50)	1 (50)
aPS/PT IgG	0	0	2 (67)	0	0	0
aD1β_2_GPI IgG	1 (11)	0	0	0	0	0

Anti-beta-2 glycoprotein-1 [aβ_2_GP1], anticardiolipin antibodies [aCL]), anti-phosphatidylserine/prothrombin antibodies (aPS/PT), anti- domain 1 of β_2_GPI (aDIβ_2_GPI).Acute disseminated encephalomyelitis [ADEM], Gllian Barre Syndrome (GBS). ‘Other’ included intracranial hypertension and a central pain syndrome. Antiphospholipid antibody overlap occurred in the following cases; Encephalopathy – one case had triple antibody positivity [aCL IgM/ aβ_2_GPI IgG/aβ_2_GPI IgA), 2 cases had double antibody positivity; aCL IgM/aβ_2_GPI IgA and aβ_2_GPI IgG/aβ_2_GPI IgA. Encephalitis - one case had triple antibody positivity; aCL IgM/ aβ_2_GPI IgG/aβ_2_GPI IgA. ADEM - one case had double antibody positivity; aCL IgM/aPS/PT IgG. Stroke - one case had double antibody positivity; aCL IgM/aβ_2_GPI IgA. GBS - one case had double antibody positivity; aCL IgM/aβ_2_GPI IgA. Other - one case had double antibody positivity; aCL IgM/aβ_2_GPI IgG. Missing data encephalopathy *n* = 1, encephalitis *n* = 1 and other *n* = 1.

Similarly, antiphospholipid antibodies were also common in the non-neurological COVID-hospitalised controls. Thirty six (76.6%) were positive for at least one antiphospholipid antibody, 22 (46.8%) positive for at least two antiphospholipid antibodies and 13 (27.7%) for three or more. The most prevalent antiphospholipid antibodies were aCL IgM (51.1%) and aB_2_GPI IgA (25.5%). These were followed by aCL IgG and IgA (21.3%), aB_2_GPI IgM (17%), aD1β_2_GPI IgG (14.9%) and aPS/PT IgM (10.6%). Out of the 47 COVID-hospitalised controls, 16 (34.0%) were positive for at least one antiphospholipid antibody in the APS criteria and 9 (19.1%) for two. A total of 25 (53.2%) patients had moderate and 9 (19.1%) had high titres.

In contrast, the prevalence of antiphospholipid antibodies in the COVID-non-hospitalised participants was considerably lower; only 14 (24.1%) were positive for aCL IgM. aPS/PT IgM (20.7%) occurred more frequently in this group compared to the others. Only 14 (48.2%) of the patients were positive for at least one antiphospholipid antibody, and 4 (13.8%) were positive for two antiphospholipid antibodies and none were triple positive. Only 9 (31.0%) patients were positive for at least one antiphospholipid antibody from the APS criteria and none for two. Out of the 29 COVID-non-hospitalised patients only 7 (24.1%) had moderate titres and no patients had high titres.

### 3.3 A distinct antiphospholipid antibody profile characterises COVID-neurological patients compared to COVID-hospitalised and non-hospitalised controls

**Fig. 1:**
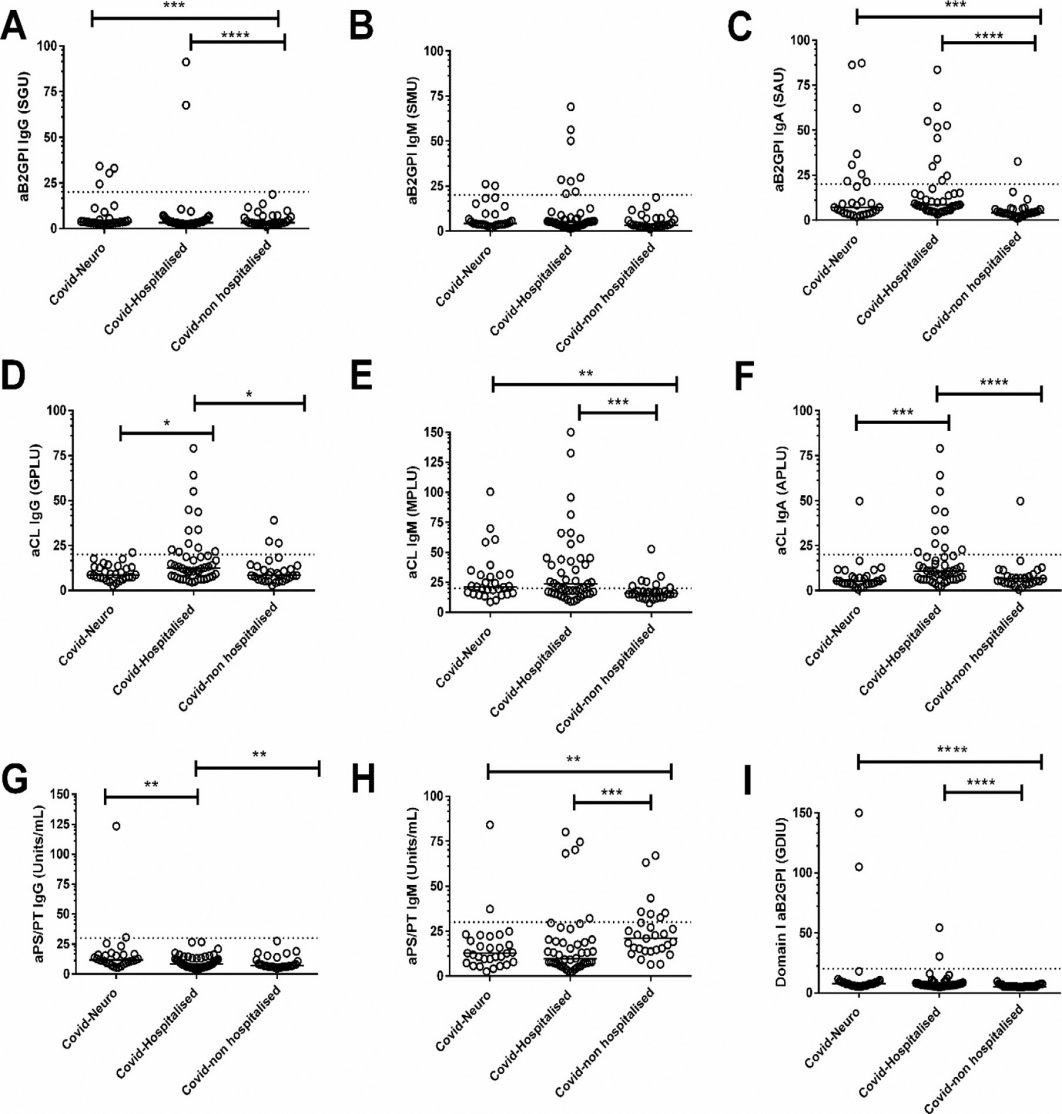
The association between antiphospholipid antibodies titres and COVID-neurological, hospitalised and non-hospitalised groups Serum samples were obtained from 106 adult participants. All were divided into the following groups; 29 COVID-non-hospitalised controls, 47 COVID-hospitalised controls, 30 COVID-neurological cases. The antiphospholipid antibody titre levels were measured across the groups for (A) anti-beta-2 glycoprotein-1 [aβ_2_GP1] IgG (B) aβ_2_GP1 IgM (C) aβ_2_GP1 IgA (D) anticardiolipin antibodies [aCL]) IgG, (E) aCL IgM, (F) aCL IgA (G) anti-Antiphosphatidylserine/prothrombin antibodies (aPS/PT) IgG, (H) aPS/PT IgM, (I) anti- domain 1 of β_2_GPI (aDIβ_2_GPI). The horizontal broken line represents the cut-off for each antiphospholipid antibodies. *P < 0.05, **P < 0.01, ***P < 0.001, and ****P < 0.0001. P values only shown when groups are significantly different. Missing data Covid-Neuro *n* = 3, Covid-Hospitalised *n* = 0, Covid non-hospitalised *n* = 0.

To understand the relationship between antiphospholipid antibodies, COVID-neurological patients, non-neurological COVID-hospitalised and non-hospitalised controls, we investigated whether there were any associations between antiphospholipid antibody titres and the different groups. We found that COVID-neurological and COVID-hospitalised patients had significantly higher levels of aB_2_GPI IgA and IgG, aCL IgM, and aB_2_GPI-D1 IgG compared to the COVID-non-hospitalised group (*p* < 0.01 in all cases, Fig. 1 and Supplement Table 2). Of these antiphospholipid antibodies, we found that the median aCL IgM met the moderate-high cut-off in both hospitalised groups.

Despite COVID-hospitalised control patients exhibiting a more severe COVID-19 illness (Table 1), there were no significant differences in titres between aβ_2_GP1 IgA (*p* = 0.152) and IgG (*p* = 0.338), aCL IgM (*p* = 0.761) and aD1β_2_GPI IgG (*p* = 0.270) compared with the COVID-neurological group (Fig. 1). However, aCL IgA (*p* = 0.001) and IgG (*p* = 0.020) titres were associated with COVID-hospitalised control group.

**Fig. 2:**
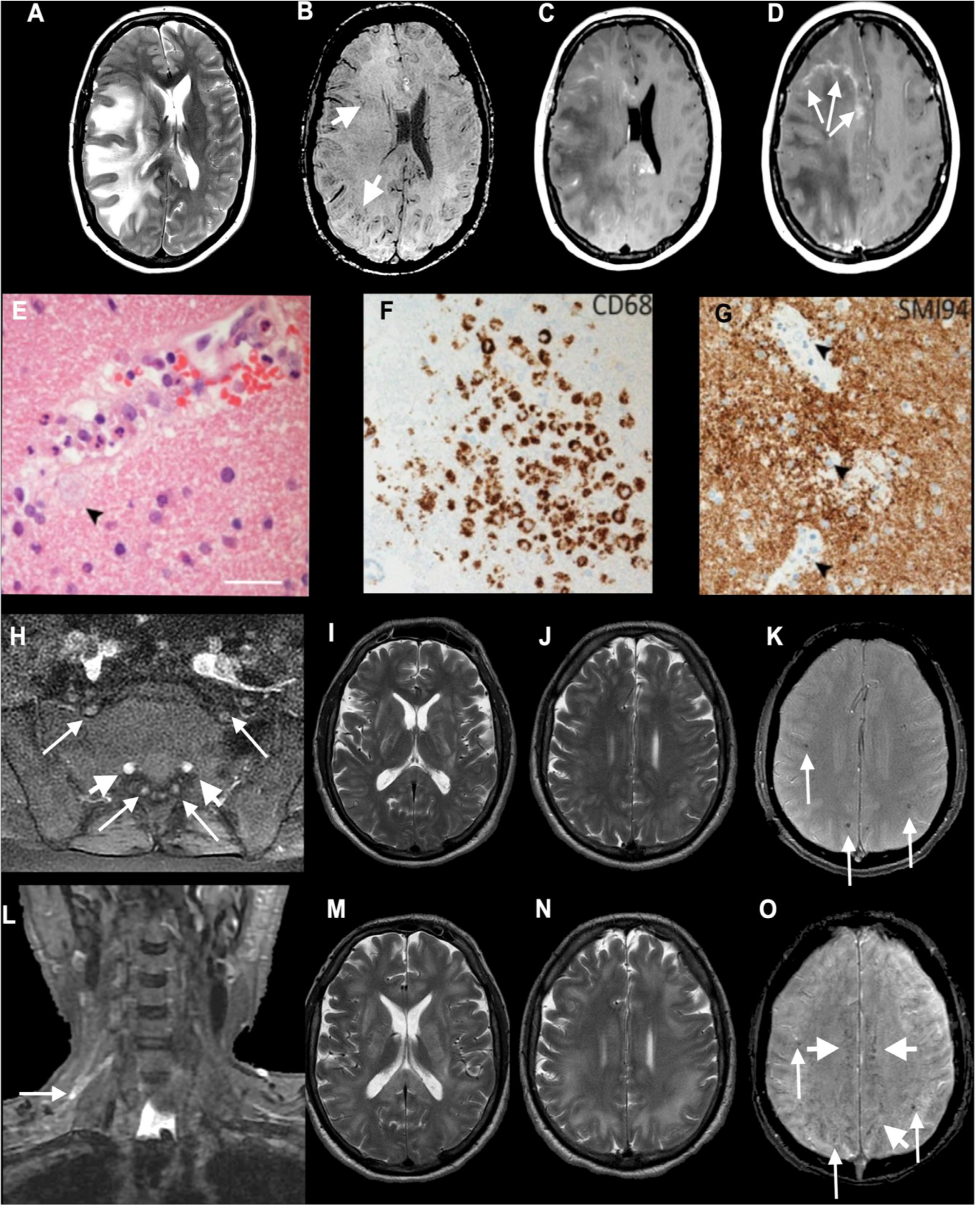
Axial MRI (A–D) and histopathology (E–G) from Patient 218, diagnosed with ADEM with a high aPS/PT IgG antibody titre (30.5 units/ml), and imaging (H–O) from Patient 218: axial T_2_-weighted (**A**), SWI (**B**), post-gadolinium (**C** and **D**) images show extensive confluent 'tumefactive' lesions involving the white matter of the right cerebral hemisphere, corpus callosum and corona radiata with mass effect, subfalcine herniation (**A**), clusters of prominent medullary veins (**B**, short arrows) and peripheral rim enhancement (**D**, arrows). (**E**) The white matter shows scattered small vessels with surrounding infiltrates of neutrophils and occasional foamy macrophages extending into the parenchyma (arrow). The endothelium is focally vacuolated but there is no evidence of vasculitis or fibrinoid vessel wall necrosis in any region. There were a few perivascular T cells in the white matter but the cortex appears normal (not shown). (**F**) CD68 stain confirms foci of foamy macrophages in the white matter, mainly surrounding small vessels. There was no significant microgliosis in the cortex (not shown). (**G**) Myelin basic protein stain (SMI94) shows areas with focal myelin debris in macrophages around vessels in the white matter (arrows) in keeping with early myelin breakdown. There is no evidence of axonal damage on neurofilament stain (not shown). Scale bars: ***E*** = 45 µm; **F** and ***G*** = 70 µm. (**H**–**O**) Patient 218; axial post-gadolinium fat-suppressed T_1_-weighted images (**H**) demonstrating pathologically enhancing extradural lumbosacral nerve roots (arrows). Note physiological enhancement of nerve root ganglia (short arrows). Coronal short tau inversion recovery (STIR) image (**L**) shows hyperintense signal abnormality of the upper trunk of the right brachial plexus (arrow). Initial axial T_2_ (**I** and **J**) and T_2_*-weighted images (**K**) show multifocal confluent T_2_ hyperintense lesions involving internal and external capsules, splenium of corpus callosum (**I**), and the juxtacortical and deep white matter (**J**), associated with microhaemorrhages (**K**, arrows). Follow-up T_2_-weighted images (**M** and **N**) show marked progression of the confluent T_2_ hyperintense lesions, which involve a large proportion of the juxtacortical and deep white matter, corpus callosum and internal and external capsules. The follow-up SWI image (**O**) demonstrates not only the previously seen microhaemorrhages (arrows) but also prominent medullary veins (short arrows). CT pulmonary angiogram excluded a pulmonary embolism but showed mild patchy ground-glass changes peripherally at the lung bases bilaterally characteristic of COVID-19 (not shown). REPRODUCED FROM PATERSON AND COLLEAGUES, BY PERMISSION OF OXFORD UNIVERSITY PRESS.

In contrast, in the COVID-neurological group aPS/PT IgG was significantly higher than both control groups (*p* < 0.01 in all cases, Fig. 1 and Supplement Table 2), indicating a degree of specificity for neurological disease. We explored the neurological subtype associated with elevated aPS/PT IgG; two cases out of three were above the moderate-high titre threshold, both with a diagnosis of acute disseminated encephalomyelitis (ADEM); case 218 is an example with accompanying histopathology (Fig. 2).

Interestingly, aPS/PT IgM was associated with COVID-non-hospitalised controls compared to the other groups (*p* < 0.01), and aB_2_GPI IgM was non-discriminatory across all groups.

### 3.4 Antiphospholipid antibodies correlate with COVID-19 disease severity markers and outcome

To consolidate our findings and gain further insight into the relationship between antiphospholipid antibodies and the COVID-neurological group, we combined all hospitalised groups to assess the potential correlation between antiphospholipid antibodies and clinical and laboratory markers of COVID-19 disease severity and outcome.

We found aPS/PT IgG titre correlated with FiO_2_ (*R*=−0.15 *p* = 0.040) but not with CRP (*R*=−0.02, *p* = 0.549) or d-dimer (*R* = 0.11, 0.649) (Table 4). In contrast, aCL IgG and IgA correlated with creatinine (aCL IgG, *R* = 0.32, *p* = 0.016; aCL IgA, *R* = 0.33, *p* = 0.005), FiO_2_ and d-dimer (aCL IgG only; R0.25, *p* = 0.011). Additionally, FiO_2_ was negatively correlated with aCL IgM (*R*=−0.25, *p* = 0.011), aβ_2_GP1 IgM (*R*=−0.14, *p* = 0.040) and aD1β_2_GPI (*R*=−0.31, *p* = 0.041). Of these antiphospholipid antibodies, aCL IgM and aD1β_2_GPI IgG titres were significantly increased in both the COVID-neurological and hospitalised groups than the COVID-non-hospitalised controls (Supplement Table 2).

**Table 4:** Correlation of antiphospholipid antibodies with clinical and laboratory variables in hospitalised patients with COVID-19.

	C-reactive protein		D-dimer		FiO_2_		Creatinine	
	*R*	*P*	*R*	*P*	*R*	*p*	*R*	*P*
aCL IgA	0.26	0.087	0.20	0.129	−0.16	**0.047**	0.33	**0.005**
aCL IgM	0.23	0.110	0.33	**0.010**	−0.25	**0.011**	0.04	0.914
aCL IgG	0.12	0.534	0.25	**0.011**	−0.21	**0.017**	0.32	**0.016**
aβ_2_GP1 IgA	0.07	0.136	0.03	0.647	−0.25	0.178	0.10	0.261
aβ_2_GP1 IgM	0.00	0.856	0.11	0.649	−0.14	**0.040**	−0.02	0.685
aβ_2_GP1 IgG	−0.19	0.787	−0.09	0.819	0.01	0.148	0.13	0.283
aPS/PT IgM	0.06	0.919	0.05	0.829	−0.22	0.116	−0.00	0.589
aPS/PT IgG	−0.02	0.549	0.11	0.755	−0.15	**0.040**	0.05	0.290
aD1β_2_GPI IgG	0.11	0.909	0.12	0.091	−0.31	0.041	−0.00	0.578

aPL antiphospholipid antibodies; aCL, anticardiolipin antibodies; aB2GPI, anti–B_2_ glycoprotein I antibodies; aPS/PT, anti-phosphatidylserine/prothrombin antibodies; aD1β2GPI, anti- Domain I B_2_ glycoprotein I antibody. FiO_2_ defines oxygen requirement. *P* < 0.05 is defined as significant (in bold).

The association between antiphospholipid antibodies and outcome measures (28-day mortality, ITU admission, venous thromboembolism, and ischaemic arterial stroke) was also assessed. Of all the antiphospholipid antibodies, raised aPS/PT IgG titre was associated with venous thromboembolism (*p* = 0.043), and raised aCL IgA titre was associated with ITU admission (*p* = 0.021) (Supplement Table 3). Increased aCL IgA (*p* = 0.020) and β_2_GP1 IgA (*p* = 0.046) was associated with not having an ischaemic stroke.

## 4 Discussion

We report the first study to examine the prevalence and pattern of antiphospholipid antibodies and associations with the neurological manifestations of COVID-19 in comparison to hospitalised and non-hospitalised COVID-19 controls. We examined nine different antiphospholipid antibodies and showed high prevalence of antiphospholipid antibody in both the COVID-neurological and COVID-hospitalised controls as compared with the COVID-non-hospitalised controls. Importantly, we made the novel observation that raised aPS/PT IgG was associated with COVID-neurological manifestation, specifically ADEM. This antiphospholipid antibody was also related to venous thromboembolism, and respiratory disease (declining oxygen requirement) in the absence of systemic inflammation (CRP). In contrast, antibodies that distinguished COVID-hospitalised controls, including aCL IgA and IgG, were associated with hypercoagulability (elevated D-dimer), respiratory and renal disease (increasing creatinine), thereby exhibiting features of the thrombo-inflammatory complications seen in COVID-19 [1,24]. Together, these results provide evidence that antiphospholipid antibodies are ubiquitous and might have a pathological role in COVID-19 and neurological manifestations of COVID-19.

Antiphospholipid antibodies are a heterogeneous group of antibodies that are important in the pathogenesis of antiphospholipid syndrome, targeting various phospholipid-binding plasma proteins such as β_2_GPI and prothrombin that bind phospholipids such as cardiolipin [5]. Their association with infections, especially viral infections, is recognised [8]. While the detection of antiphospholipid antibodies in viral infection is usually incidental, some viruses, such as hepatitis C, generate antiphospholipid antibody that is associated with thrombosis [8]. The generation of antiphospholipid antibodies in SARS-CoV-2 could be explained by molecular mimicry and neoepitope formation of the phospholipid-like epitope, via the S protein on the viral cell wall, inducing the generation of antiphospholipid antibodies [25]. Antibodies generated against these phospholipid-like proteins of SARS-CoV-2 could trigger an immunogenic response if those proteins are shared with native tissues. Notably, the generation of IgG aPS/PT cross-reacting with myelin-related protein, specifically phosphatidylserine, which is abundantly found in myelin, could potentially give rise to ADEM [9,26]. The histopathology of one of these ADEM cases demonstrated myelin-dependant pathobiology in the absence of cerebral thrombosis. In contrast, the correlation with declining oxygen requirement, trend with increasing hypercoagulability (increasing d-dimer) in the absence of systemic inflammation (CRP), is likely attributable to respiratory microvascular thrombosis [27]. The CT chest imaging of our ADEM case showed the characteristic bilateral ground-glass appearances recognised in COVID-19 [27]. Furthermore, the association with venous thromboembolism raises the possibility of co-existing thrombotic complications. Importantly, the risk of aPS/PT IgG persisting and developing antiphospholipid syndrome remains [9]. Longitudinal studies are needed, especially to ascertain clinical impact, in order to guide appropriate management.

Catastrophic antiphospholipid syndrome (CAPS) involves derangements of both inflammatory and thrombotic pathways and affects multiple organs in the body simultaneously [15]. In the CAPS registry, the most frequently affected organs were the kidneys (73%), and the lungs (60%), and the presence of aCL IgG antibody (81%) was common [28]. In our non-neurological COVID-hospitalised controls, we not only showed a modestly high rate of aCL IgG (21%) but also, an association with multiple organ disease (respiratory, renal), and hypercoagulability (raised d-dimer). While these abnormally correlated biomarkers may point to possible CAPS in the COVID-hospitalised controls, we had less evidence for this in the COVID-neurological group, who, in general, had a less severe systemic COVID-19 illness.

Moderate-high titre thresholds for antiphospholipid antibodies were similar in both the COVID-neurological group (36.6% and 20%, respectively) and COVID-hospitalised controls (53% and 19.1%, respectively) compared to the COVID non-hospitalised controls, where only 24.1% had moderate titres. Furthermore, the COVID-neurological group had similar antibody titres to hospitalised controls for aB_2_GPI IgA and IgG, aCL IgM, and aB_2_GPI-D1 IgG. IgM aCL was noteworthy, given its high prevalence of moderate-high titre thresholds. Moreover, its correlation with hypercoagulability and respiratory disease inferred a pathogenic potential. Importantly, and consistent with other reports, we showed no association between aCL IgM and large-vessel thrombosis in the pulmonary veins, deep veins or cerebral arteries [7].

The identification of a very high aD1β_2_GPI IgG (>1000unit/ml) titre is important. β_2_GPI is a crucial plasma protein in maintaining haemostasis, pathogenic antibodies to this target can result in a prothrombotic state [29]. Moreover, all domains of β_2_GPI have been described as targeted by antiphospholipid antibody, but the most clinically significant to date is antibodies to domain I [30]. The delayed presentation, in our case, is consistent with the recent description of a biphasic presentation of encephalopathy, with the delayed phase (approximately 20-days) reported as more severe [31]. The exact mechanism is unclear, and could be immune-mediated. However, the concurrent negative correlation with aD1β_2_GPI and declining oxygen requirement, raises the possibility of microvascular thrombosis in the respiratory system, which could also occur in the brain [27].

Our study has several limitations. Sample sizes were constrained by the number of cases with COVID-neurological manifestations identified during the first UK COVID-19 wave when mass hospitalisations occurred. However, in the context of this novel cohort, we identified preliminary target antiphospholipid antibodies associated with hospitalised COVID-19 and neurological manifestation of COVID-19. We did not screen for pre-morbid renal impairment, although we do not think it was prevalent, it could potentially have confounded the correlation analysis with antiphospholipid antibodies. Patients were tested for DVT or PE using doppler ultrasound and CT pulmonary angiogram respectively, if symptomatic or had persistently elevated d-dimer. There remains the possibililty of missing asymptomatic thromboembolism, but we think that this would have been in the minority. We did not have access to the required volume of citrated plasma samples needed for lupus anticoagulant testing. This would have provided additional context and risk stratification for the antiphospholipid antibody profiling results. However, we speculate that levels would have been comparable to aPS/PT as previously described [32]. For hospital-based studies, access to non-hospitalised controls is challenging. Although our non-hospitalised cohort was significantly younger and healthier, it provided a useful comparator group and the opportunity to encompass the full spectrum of COVID-19 disease severity. A non-COVID control group would have been useful to explore the relevance of antiphospholipid antibodies independently associated with mild COVID-19 disease such as aPS/PT IgM. We acknowledge that our sampling was opportunistic and not defined by a disease time point. However, we found no significant difference in the timing from COVID-19 onset to blood sampling between our hospitalised groups (data not shown). Furthermore, prophylactic or treatment dose anticoagulation was used as a prevention strategy, which could have confounded our findings. Future studies should endeavour to systematically track antiphospholipid antibodies over the full course of hospitalisation and at a 12-weeks interval, with a careful recording of drugs that interact with the clotting mechanisms.

In summary, we provide the first insight into the pathobiology of antiphospholipid antibodies in the neurological manifestation of COVID-19. We identified a high prevalence of antiphospholipid antibodies in both hospitalised groups, and found that aPS/PT IgG might be an important factor in the manifestation of ADEM, mediated by non-thrombotic mechanisms in the brain and possibly associated with thrombotic complications systemically. In contrast, aCL IgA and IgG may have a pathological role in non-neurological hospitalised patients with COVID-19. Characterisation of whether these antiphospholipid antibodies are persistent and their potential longitudinal clinical impact are required to guide appropriate management.

## Declaration of Competing Interest

LAB reports grants from GlaxoSmithKline, grants from Wellcome Trust, outside the submitted work.; RWP reports grants from Neurofilament light consortium, personal fees from GE healthcare educational talk, outside the submitted work.; CP is co-inventor for a patented Domain I-based potential therapeutic for APS.; KB reports personal fees from Abcam (advisory board / consultant), personal fees from Axon (advisory board / consultant), personal fees from Biogen (advisory board / consultant), personal fees from Julius Clinical (data monitoring committee), personal fees from Lilly (advisory board / consultant), personal fees from MagQu (advisory board / consultant), personal fees from Novartis (data monitoring committee), personal fees from Roche Diagnostic (advisory board / consultant), personal fees from Siemens Healthineers (advisory board / consultant), outside the submitted work; and is co-founder of Brain Biomarker Solutions in Gothenburg AB (BBS), which is a part of the GU Ventures Incubator Program.; CM reports personal fees from Biogen (sit on steering committee for aducanumab fees for time spent), personal fees from Roche (advisory board member fees for time spent), personal fees from Washington University (sit on therapeutic evalutation committee fees for time spent), outside the submitted work.; BS reports grants from UKRI/DHSC Global Effort on COVID-19 Research (Medical Research Council), non-financial support from UK National Institute for Health Research Global Health Research Group on Brain Infections, outside the submitted work.; MSZ reports receiving personal fees from UCB, for lecturing, outside the submitted work.; DJW reports personal fees from Bayer, personal fees from Alnylam, personal fees from Portola, outside the submitted work.; TS is on the Data Safety Monitoring Committee of Study to Evaluate the Safety and Immunogenicity of a Candidate Ebola Vaccine in Children GSK3390107A (ChAd3 EBO-Z) vaccine from GSK, is a panel member of Covid-19 Vaccine Benefit Risk Expert Working Group, which assesses the benefits and risks of Covid-19 vaccines at the Medicines and Healthcare Regulatory Agency (UK), is a member of COVID-19 Therapeutics Advisory Panel at the United Kingdom Department of Health & Social Care, and is Chair/Co-Chair of theCOVID-19 Rapid Response and Rolling Funding Initiatives, which supported development of the Oxford-Astra Zeneca Covid-19 vaccine, at the National Institute for Health Research, outside the submitted work. In addition, TS has a patent Test for bacterial meningitis based on a blood test, filed for patent pending.; HZ reports personal fees from Wave (Advisory board / consultant), personal fees from Roche Diagnostics (Advisory board / consultant), personal fees from Biogen (Sponsored lecture), personal fees (Advisory board / consultant) from Samumed, Eisai, Denali, Nervgen, Roche Diagnostics, Siemens Healthineers, Pinteon Therapeutics, AZTherapies, CogRx, personal fees (Sponsored lecture) from Alzecure, Cellectricon, and Fujirebio, outside the submitted work; and is Co-founder of Brain Biomarker Solutions in Gothenburg AB (BBS), which is a part of the GU Ventures Incubator Program.; JMS reports work support from National Institute for Health Research University College London Hospitals Biomedical Research Centre, during the conduct of the study; personal fees from Roche Pharmaceuticals (consulting), personal fees from Eli Lilly (consulting, lecturing), Drug Safety Monitoring Board at Axon Neuroscience, non-financial support from AVID Radiopharmaceuticals (PET tracer provision), consulting on educational activities from Biogen, personal fees from Merck (consulting), royalties from Oxford University Press, royalties from Henry Stewart Talks, grants from Wolfson Foundation, grants from Engineering and Physical Sciences Research Council (EP/J020990/1), grants from Medical Research Council Dementias Platform UK (MR/L023784/1), grants from Alzheimer's Research UK (ARUK-Network 2012-6-ICE; ARUK-PG2017-1946; ARUK-PG2017-1946), grants from Brain Research UK (UCC14191), grants from Weston Brain Institute (UB170045), grants from European Union's Horizon 2020 research and innovation programme (Grant 666,992), grants from British Heart Foundation (PG/17/90/33,415), personal fees from Alzheimer's Research UK (Chief Medical Officer), personal fees from UK Dementia Research Institute (Medical Advisor), outside the submitted work.; HC reports institutional research support and support to attend scientific meetings, and honoraria for lectures paid to UCLH charities from Bayer Healthcare, and consultancy fees paid to University College London Hospitals Charity from UCB Biopharma, outside the submitted work.; All other authors have nothing to disclose.

## Appendix Supplementary materials

Image, application 1

Image, application 2
