# Supplementary material for: Antiphospholipid antibodies and neurological manifestations in acute COVID-19: A single-centre cross-sectional study
Source: eClinicalMedicine. 2021 Aug 12;39:101070. doi: 10.1016/j.eclinm.2021.101070 (PMC8358233; doi:10.1016/j.eclinm.2021.101070)
Supplement: Supplementary file 1 [file mmc1.docx]

**Supplement Table 1:** Clinical characteristic of a patient with very high aD1β_2_GPI IgG titre (˃1000 units/mL)

|  | **Case 1**  **(COV_028)** |
| --- | --- |
| **Diagnosis** | Encephalopathy |
| **Clinical history** | Mild respiratory illness  One month later, presented to the hospital with an amnestic syndrome.  MRI imaging – equivocal high signal of both hippocampi on FLAIR, but heavily degraded by movement artefact.  Pulsed IV methyl prednisolone followed by 6 weeks high dose oral steroids; incomplete recovery. Remained cognitively impaired requiring care with activities of daily living. |
| **Ethnicity** | Non-white |
| **Age** | 68 |
| **Sex** | Male |
| **ECDC classification of COVID-19** | Confirmed |
| **SARS CoV 2 serology positive** | Yes |
| **WHO COVID-19 disease severity** | Mild/moderate |
| **Other aPL positivity** | aCL IgM (100 units/ml) |
| **Co-morbidities** | nil |
| **Baseline D-dimer (g/DL)** | 10,000 |
| **Baseline CRP (mmol/L)** | 192 |
| **CSF** | WCC 1, RCC 104, Protein 0.4, Glucose 3.7,Oligoclonal bands negative |
| **VTE** | No |
| **LMWH** | No |
| **ITU admission** | No |
| **28-day survival** | Alive |
| aPL – antiphospholipid antibody; CSF- cerebrospinal fluid; VTE – venous thromboembolism; LMWH – low molecular weight heparin. | |

**Supplement Table 2.** antiphospholipid antibody titres among COVID neurological and non-neurological groups

|  | COVID-Neuro  n=30  Median (IQR) | COVID-Hospitalised  n=47  Median (IQR) | COVID-non hospitalised  n=29  Median (IQR) | p |
| --- | --- | --- | --- | --- |
| aβ_2_GPI IgG | 3.5 (3.0, 5.5) | 3.2 (2.8, 4.8) | 1.8 (1.5, 1.9) | COVID-neuro v COVID-hosp=0.338  COVID-neuro v non-COVID-hosp=**0.000**  COVID-hosp v non-COVID-hosp=**0.001**  All=**0.001** |
| aβ_2_GPI IgM | 4.2 (3.6, 7.5) | 5.0 (3.0, 8.8) | 3.2 (2.7, 7.0) | COVID-neuro v COVID-hosp=0.761  COVID-neuro v non-COVID-hosp=0.191  COVID-hosp v non-COVID-hosp=0.271  All= 0.404 |
| aβ_2_GPI IgA | 7.1 (3.8, 21.4) | 8.6 (5.6, 22) | 4.1 (3.2, 6.1) | COVID-neuro v COVID-hosp=0.152  COVID-neuro v non-COVID-hosp=**0.011**  COVID-hosp v non-COVID-hosp=**0.000**  All=**0.000** |
| aCL IgG | 8.7 (7.2, 12.9) | 12.3 (7.3, 21.30) | 8.5 (6.2, 13.4) | COVID-neuro v COVID-hosp=**0.020**  COVID-neuro v non-COVID-hosp=0.994  COVID-hosp v non-COVID-hosp=**0.037**  All=**0.030** |
| aCL IgM | 21.3 (15.8, 31.2) | 23.8 (15.5, 45) | 16.0 (12.5, 17.5) | COVID-neuro v COVID-hosp=0.521  COVID-neuro v non-COVID-hosp=**0.008**  COVID-hosp v non-COVID-hosp=**0.001**  All=**0.003** |
| aCL IgA | 5.4 (3.8, 7.8) | 10.8 (6.2, 19.3) | 6.6 (4.3, 8.0) | COVID-neuro v COVID-hosp=**0.001**  COVID-neuro v non-COVID-hosp=**0.405**  COVID-hosp v non-COVID-hosp=**0.000**  All=**0.000** |
| aPS/PT IgG | 11.8 (9.2,16.1) | 8.1 (5.8, 13.2) | 6.9 (6.0, 10.6) | COVID-neuro v COVID-hosp=**0.007**  COVID-neuro v non-COVID-hosp=**0.002**  COVID-hosp v non-COVID-hosp=**0.008**  All=**0.000** |
| aPS/PT IgM | 12.8 (7.8, 22.4) | 12.7 (7.6-20.9) | 21 (14.7, 29.7) | COVID-neuro v COVID-hosp=0.152  COVID-neuro v non-COVID-hosp=**0.007**  COVID-hosp v non-COVID-hosp=**0.001**  All=0.1205 |
| aD1β_2_GPI IgG | 7.5 (6.1, 9.0) | 6.6 (5.9, 8.2) | 5.2 (5.2, 6.7) | COVID-neuro v COVID-hosp=0.270  COVID-neuro v non-COVID-hosp=**0.000**  COVID-hosp v non-COVID-hosp=**0.000**  All= **0.000** |
| aPL antiphospholipid antibodies; aCL, anticardiolipin antibodies; aB2GPI, anti–B_2_ glycoprotein I antibodies; aPS/PT, anti-phosphatidylserine/prothrombin antibodies; aD1β2GPI IgG, anti- Domain I B_2_ glycoprotein I antibody. P<0.05 is defined as significant (in bold). | | | | |

**Supplement Table 3:** Associations with antiphospholipid antibody titres and outcome in COVID-19 hospitalised patients

| Outcome | 28-day mortality | | | ITU admission | | | Venous Thromboembolism | | | Ischaemic arterial stroke | | | |
| --- | --- | --- | --- | --- | --- | --- | --- | --- | --- | --- | --- | --- | --- |
| Median: IQR | **Yes** | **No** | **P** | **Yes** | **No** | **P** | **Yes** | **No** | **p** | **Yes** | **No** | **P** |  |
| aCL IgA | 7.0  (4.4, 9.9) | 8.0  (5.4,14.7) | 0.248 | 10.1  (6.0,19.0) | 6.6  (4.0, 11.1) | **0.021** | 11.3  (5.7, 38.6) | 7.4  (4.9, 13.5) | 0.227 | 4.2  (3.8, 5.4) | 8.5  (6.0, 15.6) | **0.020** |  |
| aCL IgM | 21.8  (18.2,45.3) | 21.9  (16.1,39.4) | 0.924 | 24.7  (18.1,45.3) | 20.7  (15.5, 32.1) | 0.206 | 23.5  (18.1, 36.9) | 22.0  (16.1, 39.8) | 0.803 | 21.1  (16.1, 24.3) | 22.0  (16.1, 41.2) | 0.566 |  |
| aCL IgG | 8.6  (6.2,12.7) | 12.0  (7.3,17.5) | 0.104 | 12.6  (7.0,20.4) | 9.1  (7.2, 13.5) | 0.174 | 14.4  (10.2, 38.6) | 10.2  (6.8, 15.2) | 0.082 | 8.7  (5.5, 10.4) | 11.1  (7.3, 17.2) | 0.132 |  |
| aβ_2_GP1 IgA | 8.3  (4.5,34.0) | 8.4  (5.4,21.4) | 0.903 | 8.9  (6.4,23.3) | 8.4  (5.1, 21.4) | 0.378 | 9.1  (5.0, 22.4) | 8,3  (5.2, 21.7) | 0.948 | 4.3  (3.4, 8.3) | 9.1  (5.6, 21.8) | **0.046** |  |
| aβ_2_GP1 IgM | 5.0  (3.5,12.5) | 5.0  (3.4,8.8) | 0.801 | 5.0  (3.2,8.2) | 4.7  (3.6, 10.6) | 0.860 | 4.9  (3.6, 5.5) | 5.0  (3.4, 9.6) | 0.833 | 4.3  (3.5, 13.8) | 4.8  (3.4, 8.2) | 0.976 |  |
| aβ_2_GP1 IgG | 3.2  (2.8, 3.7) | 3.5  (2.9,5.5) | 0.153 | 3.5  (3.1,4.8) | 3.5  (2.8, 5.0) | 0.695 | 3.4  (2.9, 5.3) | 3.6  (2.9, 4.4) | 0.864 | 3.3  (3.0, 3.8) | 3.5  (2.8, 4.8) | 0.890 |  |
| aPS/PT IgM | 17.6  (5.3, 27.1) | 11.9  (7.5,19.3) | 0.555 | 13.1  (7.1,20.2) | 11.4  (7.5, 18.5) | 0.480 | 17.6  (12.6, 22.1) | 11.9  (7.1, 19.3) | 0.198 | 12.6  (5.4, 22.1) | 12.6  (7.3, 19.3) | 0.733 |  |
| aPS/PT IgG | 11.3  (5.6,13.3) | 10.8  (7.3, 14.5) | 0.604 | 11.3  (14.5,14.5) | 10.4  (6.4, 15.2) | 0.871 | 13.3  (9.2, 16.2) | 9.6  (6.2, 13.1) | **0.043** | 9.2  (5.6, 12.1) | 11.0  (6.4, 15.2) | 0.287 |  |
| aD1β_2_GPI IgG | 6.7  (5.8,7.8) | 7.0  (6.0,9.3) | 0.488 | 6.9  (5.9,8.7) | 7.0  (6.0, 8.6) | 0.652 | 7.2  (5.9, 8.7) | 6.7  (5.9, 8.6) | 0.652 | 7.7  (6.3, 8.6) | 6.8  (5.9, 8.7) | 0.514 |  |
| aPL antiphospholipid antibodies; aCL, anticardiolipin antibodies; aB2GPI, anti–B_2_ glycoprotein I antibodies; aPS/PT, anti-phosphatidylserine/prothrombin antibodies; aD1β2GPI IgG, anti- Domain I B_2_ glycoprotein I antibody. | | | | | | | | | | | | |  |
