# Supplementary material for: Antiphospholipid antibodies and neurological manifestations in acute COVID-19: A single-centre cross-sectional study
Source: eClinicalMedicine. 2021 Aug 12;39:101070. doi: 10.1016/j.eclinm.2021.101070 (PMC8358233; doi:10.1016/j.eclinm.2021.101070)
Supplement: Supplementary file 2 [file mmc2.docx]

**The UCLH Queen Square COVID-19 Biomarker Study group**

1. Patricia McNamara

National Hospital for Neurology and Neurosurgery, University College London Hospitals NHS Foundation Trust, Queen Square, London, UK

1. Selina Edwards

National Hospital for Neurology and Neurosurgery, University College London Hospitals NHS Foundation Trust, Queen Square, London, UK

1. Roberto Macarimban-Inglesant

National Hospital for Neurology and Neurosurgery, University College London Hospitals NHS Foundation Trust, Queen Square, London, UK

1. Isabella Maritnez

National Hospital for Neurology and Neurosurgery, University College London Hospitals NHS Foundation Trust, Queen Square, London, UK

1. Bhagteshwar Singh

National Institute for Health Research Health Protection Research Unit in Emerging and Zoonotic Infections, Institute of Infection, Veterinary and Ecological Sciences, University of Liverpool, UK

1. Rubika Balendra

National Hospital for Neurology and Neurosurgery, University College London Hospitals NHS Foundation Trust, Queen Square, London, UK

UCL Queen Square Institute of Neurology, London, UK

1. Guru Kumar

Darent Valley Hospital, Dartford, Kent, UK

1. Soon Tjin Lim

National Hospital for Neurology and Neurosurgery, University College London Hospitals NHS Foundation Trust, Queen Square, London, UK

1. Laura Zambreanu

National Hospital for Neurology and Neurosurgery, University College London Hospitals NHS Foundation Trust, Queen Square, London, UK

UCL Queen Square Institute of Neurology, London, UK

Watford General Hospital, Watford, Hertfordshire, UK

1. Maria Thom

UCL Queen Square Institute of Neurology, London, UK

National Hospital for Neurology and Neurosurgery, University College London Hospitals NHS Foundation Trust, Queen Square, London, UK
